# Myelination Increases the Spatial Extent of Analog-Digital Modulation of Synaptic Transmission: A Modeling Study

**DOI:** 10.3389/fncel.2020.00040

**Published:** 2020-03-03

**Authors:** Mickaël Zbili, Dominique Debanne

**Affiliations:** ^1^Lyon Neuroscience Research Center, INSERM U1028-CNRS UMR5292-Université Claude Bernard Lyon1, Lyon, France; ^2^UNIS UMR 1072 INSERM, AMU, Marseille, France

**Keywords:** myelin, axon, axonal space constant, analog digital facilitation, spike shape, ion channels, axonal length constant

## Abstract

Analog-digital facilitations (ADFs) have been described in local excitatory brain circuits and correspond to a class of phenomena describing how subthreshold variations of the presynaptic membrane potential influence spike-evoked synaptic transmission. In many brain circuits, ADFs rely on the propagation of somatic membrane potential fluctuations to the presynaptic bouton where they modulate ion channels availability, inducing modifications of the presynaptic spike waveform, the spike-evoked Ca^2+^ entry, and the transmitter release. Therefore, one major requirement for ADFs to occur is the propagation of subthreshold membrane potential variations from the soma to the presynaptic bouton. To date, reported ADFs space constants are relatively short (250–500 μm) which limits their action to proximal synapses. However, ADFs have been studied either in unmyelinated axons or in juvenile animals in which myelination is incomplete. We examined here the potential gain of ADFs spatial extent caused by myelination using a realistic model of L5 pyramidal cell. Myelination of the axon was found to induce a 3-fold increase in the axonal length constant. As a result, the different forms of ADF were found to display a much longer spatial extent (up to 3,000 μm). In addition, while the internodal length displayed a mild effect, the number of myelin wraps ensheathing the internodes was found to play a critical role in the ADFs spatial extents. We conclude that axonal myelination induces an increase in ADFs spatial extent in our model, thus making ADFs plausible in long-distance connections.

## Introduction

Analog-digital facilitation (ADF) is a context-dependent modulation of synaptic transmission reported in local excitatory circuits (Alle and Geiger, 2006; Shu et al., 2006; Kole et al., 2007; Sasaki et al., 2012; Debanne et al., 2013; Bialowas et al., 2015; Rama et al., 2015; Zbili and Debanne, 2019). To date, two major types of ADF have been described: depolarization-induced analog-digital facilitation (d-ADF) and hyperpolarization-induced analog-digital facilitation (h-ADF). In cortical circuits, d-ADF is an enhancement of the spike-evoked synaptic transmission following a long (3–10 s) subthreshold depolarization of the presynaptic cell. The mechanism underlying d-ADF in cortical networks relies on Kv1 channel inactivation. The subthreshold depolarization leads to inactivation of axonal Kv1 channels which provokes an increase in the presynaptic spike duration (spike broadening), an increase in the spike-evoked Ca^2+^ entry in the presynaptic bouton and an enhancement of the transmitter release (reviewed in Debanne et al., 2013; Zbili and Debanne, 2019). h-ADF is a much faster process relying on the recovery of axonal Nav channels from inactivation (or deactivation). A short hyperpolarization of the presynaptic cell (15–200 ms) leads to the deactivation of axonal Nav channels, which provokes an increase in the presynaptic spike amplitude, an increase in the spike-evoked Ca^2+^ entry and an enhancement of synaptic transmission. Importantly, for d-ADF and h-ADF to occur at a specific synapse, membrane potential variations of the soma (depolarization or hyperpolarization) have to propagate to the presynaptic bouton to impact the local spike shape (duration or amplitude). Therefore, the spatial extent of d-ADF and h-ADF and the number of synapses impacted by these phenomena are mainly determined by the axonal length constant. By increasing the axonal membrane resistance, myelination could increase the axonal length constant (Castelfranco and Hartline, 2015; Alcami and El Hady, 2019) and therefore expand the number of postsynaptic cells impacted by ADFs. The space constant of analog-digital modulation in unmyelinated axons has been shown to vary between 145 and 430 μm depending of the nature of the subthreshold signal used to induce ADF, the number of branch points, and the cell type (Alle and Geiger, 2006; Shu et al., 2006; Sasaki et al., 2012; Bialowas et al., 2015; Rama et al., 2015). In L5 pyramidal neurons which display a myelinated main axon and unmyelinated collaterals, the length constant of the main axon has been evaluated from 417 μm to 1,180 μm (Shu et al., 2006; Kole et al., 2007; Christie and Jahr, 2009; Cohen et al., 2020). We hypothesized that the development of myelin sheaths in L5 pyramidal neurons, which occurs mainly between P10 and P25 in rats (Battefeld et al., 2019), should increase the axonal length constant and therefore increase the spatial extent of ADFs.

Using a computational approach, we show here that myelination increases the axon length constant by a factor 3 leading to ADFs expression more than 2 mm away from the soma. In addition, the number of myelin layers wrapping the internodes were found to have a critical impact on ADF spatial extent.

## Materials and Methods

### Model Morphology

A multi-compartment model of a 36 days-old rat L5 pyramidal neuron was simulated with NEURON 7.6 (see Supplementary Figure S1 for model morphology). The neuronal morphology was taken from a reconstructed neuron by Hay et al. (2011) available on Neuromorpho.org (Ascoli et al., 2007; Neuromorpho ID: NMO_07763; Neuron Name: C080418A-1-SR). The dendritic tree of this neuron was fully reconstructed while the axonal tree was partially reconstructed (up to 1 mm from the soma). The neuron is composed of a dendritic tree, a soma, an axon hillock, an axon initial segment (AIS) and an axonal tree. The axonal tree is composed of the main axon presenting a diameter of 1.14 μm and six axonal collaterals with a diameter of 0.23 μm. The collaterals connect to the main axon at 128.5 μm, 129.6 μm, 300.9 μm, 301.5 μm, 810.1 μm, and 993.9 μm from the soma. We kept unchanged the morphology except for the axon. In fact, in order to observe the spatial extent of ADF on distal synapses, we extended the main axon (total length in our model: 20 mm) and added four distant collaterals branching the main axon 1,907.4, 2,922.4, 3,937.4 and 4,952.4 μm from the soma. Presynaptic sites containing presynaptic Ca^2+^ channels were placed every 8 μm into the axon collaterals (Romand et al., 2011) leading to a total number of 1,982 presynaptic sites into the model. The axial intracellular resistivity was fixed to 150 Ωcm in all the model compartments. The membrane capacitance was fixed to 1 μF/cm^2^ in all the model compartments except for myelin sheaths (see below). All simulations were run with 6.25 μs time steps and the nominal temperature of the simulation was 37°C. The measurement locations for subthreshold voltage fluctuations and AP waveform were the nodes of Ranvier of the main axon (or the equivalent places in the case of unmyelinated and hybrid models). The measurement locations for spike evoked Ca^2+^ entry and synaptic transmission were the presynaptic sites located in the axon collaterals, leading to an evaluation of d-ADF and h-ADF at these presynaptic sites.

### Ionic Conductance

The model contains six types of conductance: leak channels, potassium delay rectifier channels (Kdr), Kv1 channels, Nav1.2 channels, Nav1.6 channels, and P/Q-type calcium channels. The biophysics of Kdr, Nav1.2, and Nav1.6 were taken from Hu et al. (2009), the biophysics for Kv1 channels were taken from Shu et al. (2007b) and the biophysics for P/Q type calcium channels were taken from Bischofberger et al. (2002). The equilibrium potentials for Na^+^, K^+^, Ca^2+^ were set respectively to +60 mV, −90 mV and +140 mV.

In all the simulations, the densities of the different channels in the dendrites, the soma, the axonal hillock, and the AIS were taken from a previously published model of L5 pyramidal neurons (Hu et al., 2009; see Table 1). These densities were unchanged in the different simulations. In contrast, the channel densities in the axon were modified according to the presence of myelin, the length of the internodes and the number of myelin wraps. These modifications of channel density were made in order to preserve the axonal spike waveform at resting membrane potential measured in the middle of the axon (10 mm away from the soma; Supplementary Figure S2). The preservation of the basal axonal spike waveform in the different simulations was mandatory in order to compare the various conditions we studied. In fact, ADFs depend on spike shape modifications and are highly sensitive to the basal spike waveform. The densities of axonal channels in the different simulations are specified in Table 2.

**Table 1:** The density of channels in dendrites, soma, axonal hillock, and axon initial segment (AIS).

	Dendrites	Soma	Axon Hillock	AIS
Leak conductance (pS/μm^2^)	0.333	0.333	0.333	0.333
Leak channels reversal potential (mV)	−69.5	−69.5	−69.5	−69.5
Nav1.2 conductance (pS/μm^2^)	80	80	2,560	3,072–0
Nav1.6 conductance (pS/μm^2^)	0	0	0	128–1,920
K_DR_ conductance (pS/μm^2^)	10	20	100	100

**Table 2:** Parameters of the different axonal simulations.

		Non-myelinated axon	Hybrid axon	Myelinated axon 1	Myelinated axon 2	Myelinated axon 3	Myelinated axon 4	Myelinated axon 5	Myelinated axon 6	Myelinated axon 7
Mean Internodes length (μm)		~100	~100	~100	~50	~25	~100	~100	~100	~100
Myelin wraps number		0	0	15	15	15	2	5	10	20
Myelin conductance (pS/cm^2^)		/	/	0.333 ÷ 30	0.333 ÷ 30	0.333 ÷ 30	0.333 ÷ 4	0.333 ÷ 10	0.333 ÷ 20	0.333 ÷ 40
Myelin capacitance (μF/cm^2^)		/	/	1 ÷ 30	1 ÷ 30	1 ÷ 30	1 ÷ 4	1 ÷ 10	1 ÷ 20	1 ÷ 40
Axonal leak conductance (pS/μm^2^)		0.333	0.333	0.333	0.333	0.333	0.333	0.333	0.333	0.333
Axonal capacitance (μF/cm^2^)		1	1	1	1	1	1	1	1	1
Axonal leak channels reversal potential (mV)		−38.3	−38.3	−38.3	−38.3	−37.2	−38	−38.3	−38	−37.8
Nav1.6 conductance (pS/μm^2^)	internodes	370	370	0	0	0	0	0	0	0
	nodes	370	1,184.4	1,184.4	757.9	567.1	6016	2,757.7	1,590.5	987.4
Kv1 conductance (pS/μm^2^)	internodes	26.8	26.8	0	0	0	0	0	0	0
	nodes	26.8	85.5	85.5	56.3	43.1	393	191.5	113.5	71.8
P/Q type conductance (pS/μm^2^)	internodes	0	0	0	0	0	0	0	0	0
	nodes	1	1	1	1	1	1	1	1	1

### Myelin Modeling

To model the myelin sheath, one must consider that one passive plasma membrane can be considered as a parallel RC circuit. To simulate the myelin sheath, we used the “extracellular” mechanism of the Neuron 7.6 software into the internodes of the model. This mechanism adds a layer of RC circuit to the internodes (Supplementary Figure S3). The passive conductance G_my_ and the capacitance C_my_ of this added layer depend on the number of myelin wraps. We assumed that the passive membrane conductance and the membrane capacitance of the myelin plasma membrane are equal to those of the axon, i.e., G_ax_, and C_ax_. As one myelin wrap is composed of two myelin membranes, a myelin sheath composed of one myelin wrap presents a conductance G_my_ = G_ax_/2 and a capacitance C_my_ = C_ax_/2. This reasoning extended to a myelin sheath composed of n myelin wraps gives G_my_ = G_ax_/(2*n) and C_my_ = C_ax_/(2*n). To evaluate the number of myelin wraps we used a g-ratio of 0.698 which has been evaluated in L5 pyramidal neurons of adult rats (Cohen et al., 2020), the diameter of the main axon (1.14 μm), the thickness of the periaxonal space (12.3 nm; Cohen et al., 2020) and the thickness of one plasma membrane (7.5 nm). The g-ratio is the ratio of the inner axonal diameter to the total outer diameter. Therefore, $g=\frac{r_{ax}}{r_{ax}+t_{p}+t_{my}}$, where g is the g-ratio, rax is the axonal radius, tp is the periaxonal space thickness and tmy is the myelin sheath thickness. From this equation, we can see that: $t_{my}=r_{ax}*\left(\frac{1}{g}-1\right)-t_{p}$. With a g of 0.698, r_ax_ of 0.57 μm and t_p_ of 0.0123 μm, we obtained t_my_ = 0.234 μm. Assuming a thickness of 7.5 nm for one plasma membrane, we obtained that the myelin sheath is constituted of 31.2 plasma membranes. As one myelin wrap is constituted of two plasma membranes, we concluded that the myelin sheath is composed of 15.6 myelin wraps. We chose to apply a value of 15 myelin wraps in our main model of myelinated axon which is in the range of the values observed with electron microscopy (Cohen et al., 2020). Consequently, G_my_ = G_ax_/30 and C_my_ = C_ax_/30 in our main model of a myelinated axon. The values of myelin conductance and myelin capacitance in the different simulations are listed in Table 2.

### Postsynaptic Responses

To obtain the postsynaptic responses, we used Alpha Synapse Point Processes from Neuron 7.6 inserted into postsynaptic cells. The weights of the synapses were calculated using the charge of the spike-evoked Ca^2+^ entry in the presynaptic sites with the following power law:

*W* = A_*_ (*Q*_*Ca*_^2+^)^2.5^

where W is the synaptic weight, A is a scaling factor and Q_Ca_ is the charge of the spike-evoked Ca^2+^ current (Scott et al., 2008). Therefore, an increase in the Ca^2+^ entry produced by an increase in presynaptic spike amplitude or duration led to an increase in the postsynaptic response amplitude.

### Current Injection

AP was produced by a 3 nA current during 3 ms. The 15 mV subthreshold depolarization was produced by a 322 pA current during 10 s. The 15 mV hyperpolarization was produced by a −344 pA current for 200 ms. All these currents were injected into the soma.

### Space Constants Calculation

The different phenomena we observed (subthreshold depolarization, subthreshold hyperpolarization, depolarization-induced AP area increase, hyperpolarization-induced AP overshoot increase) propagated decrementally into the main axon. Due to the presence of axon collaterals and myelinated internodes, the propagations did not follow exactly a monoexponential decay, as it would be expected if the axon was a simple cable. However, in previously published experimental studies, the space constants were evaluated using fit with monoexponential decaying functions. Therefore, in order to compare the values obtained into our model and the values found in previous experimental studies, we chose to define the space constant as the distance at which a given phenomenon reaches 37% of its original value. For example, when we depolarized the soma by 15 mV, we defined the space constant of subthreshold depolarization propagation as the axonal point where the depolarization has decreased to the value of 5.55 mV (15*0.37 = 5.55). Importantly, in some conditions, the phenomena did not display a monotonic decay into the axon but evolved in a biphasic manner. We chose to not provide any space constant measurement for these cases.

## Results

In order to evaluate the impact of axonal myelination on ADFs spatial extent, we used a computational approach. We used a reconstructed morphology of an L5 pyramidal neuron from a young adult rat (Hay et al., 2011, see “Materials and Methods” section for details). The main axon is partially reconstructed (up to 1 mm from the soma) and present six collaterals located respectively at 128.5 μm, 129.6 μm, 300.9 μm, 301.5 μm, 810.1 μm, and 993.9 μm from the soma. In order to observe the propagation of ADFs to long-range connections, we extended the main axon (up to 20 mm from the soma) and we added four distant collaterals located 1,907.4, 2,922.4, 3,937.4 and 4,952.4 micrometers from the soma (the distant collaterals were 1 mm length). Supplemental parts diameters had the same values as the original axon described in Hay et al. (2011): 1.14 μm for the main axon and 0.23 μm for the collaterals. In our model, dendrites, soma and axonal hillock contained Nav1.2 channels, potassium delay-rectifier channels (Kv) and leak channels. AIS contained Nav1.2 channels, Nav1.6 channels, potassium delay-rectifier channels (Kv) and leak channels. The axon contained Nav1.6 channels, Kv1 channels and leak channels. To evaluate the effect of spike waveform on synaptic transmission, the axon collaterals also displayed presynaptic sites every 8 μm (Romand et al., 2011) which, contained a weak density of P/Q type calcium channels (1 pS/μm^2^). Given the axonal tree extension, the model presented 1,982 presynaptic sites distributed all along the axon collaterals. Spike-evoked Ca^2+^ entry and corresponding postsynaptic responses were computed at these presynaptic sites (see “Materials and Methods” section). In all our simulations, the densities of channels in the dendrites, the soma, the axon hillock and the AIS were kept unchanged and were taken from a previous model of L5 pyramidal neurons (Hu et al., 2009; Table 1). We only modified the reversal potential of leak channels (E_leak_ = −69.5 mV) to obtain a resting membrane potential of −70 mV.

In a first step, we simulated a non-myelinated axon. In this model, the densities of Nav1.6 and Kv1 channels are homogenous all along the axonal tree (g_Na1.6_ = 370 pS/μm^2^ and g_Kv1_ = 26.8 pS/μm^2^; Table 2: Non-Myelinated axon). The density of leak channels was homogeneous all along the axonal tree and equal to its value in other compartments (0.333 pS/μm^2^). However, in order to maintain the resting potential at −70 mV, we had to fix E_leak_ at −38.3 mV in the axon.

### Both d-ADF and h-ADF Are Expressed in the Model

**Figure 1:**
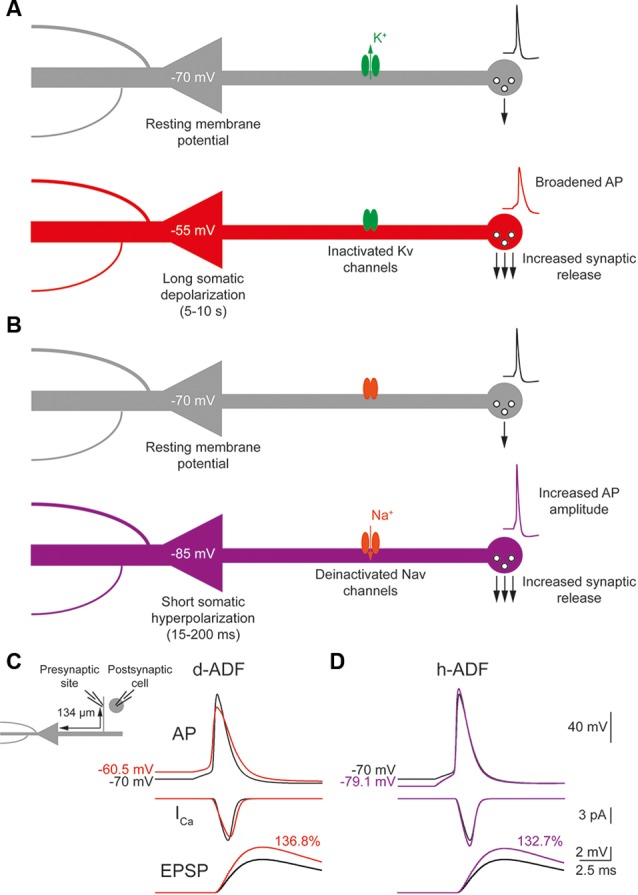
Depolarization-induced analog digital facilitation (d-ADF) and hyperpolarization-induced analog digital facilitation (h-ADF) are expressed in the model. **(A)** Schematic description of the d-ADF mechanism. A long somatic depolarization provokes the inactivation of axonal Kv1 channels leading to a broadening of the presynaptic spike and an increase in the transmitter release. **(B)** Schematic description of the h-ADF mechanism. A brief somatic hyperpolarization provokes the deinactivation of axonal Nav channels leading to an increase in both the presynaptic spike amplitude and the transmitter release. **(C)** d-ADF is present in the model. A spike generated from the depolarized Vm (red trace) is broader than the spike generated from the resting membrane potential (black trace). Therefore, the spike generated from a depolarized Vm leads to a larger presynaptic Ca^2+^ entry and a bigger EPSP in the postsynaptic cell. Note that the depolarization is induced by somatic current injection and that the traces were recorded in a presynaptic site, 134 μm from the soma. **(D)** h-ADF is present in the model. A spike generated from the hyperpolarized Vm (purple trace) presents a larger amplitude than the spike generated from the resting membrane potential (black trace). Therefore, the spike generated from a hyperpolarized Vm leads to a larger presynaptic Ca^2+^ entry and a bigger EPSP in the postsynaptic cell. Note that the hyperpolarization is induced by somatic current injection and that the traces were recorded in a presynaptic site, 134 μm from the soma.

The d-ADF occurs when the presynaptic neuron is depolarized before spike generation leading to an increase in transmitter release (Figure 1A). In pyramidal neurons, d-ADF is a slow process needing several seconds of depolarization to produce an increase in synaptic transmission (Shu et al., 2006; Kole et al., 2007; Sasaki et al., 2012; Bialowas et al., 2015). This slow time-constant is explained by the slow inactivation time-constant of Kv1 channels (1,500 ms; Shu et al., 2007b). In fact, d-ADF is a Kv1-dependent mechanism in pyramidal neurons: the subthreshold depolarization entails the inactivation of axonal Kv1 channels, leading to the broadening of the axonal spike, an increase in spike-evoked Ca^2+^ entry at the presynaptic bouton and an increased transmitter release (Figure 1A). Importantly, the subthreshold depolarization entails also the inactivation of axonal Nav channels leading to a decrease in the axonal spike amplitude (Shu et al., 2006). However, the interplay between the spike broadening and the decrease in spike amplitude leads to an overall increase in spike area leading to an enhancement of presynaptic Ca^2+^ entry and transmitter release (Shu et al., 2006; Kole et al., 2007; Bialowas et al., 2015). To verify the presence of d-ADF in the model, we injected current into the soma in order to produce a spike either generated from the resting membrane potential (V_m_ = −70 mV) or after a 10 s subthreshold depolarization of the soma at −55 mV. Then, we measured the subthreshold depolarization value, the spike waveform, the spike-evoked-calcium entry and the postsynaptic response amplitude at the first presynaptic site (located 134 μm from the soma). We found that a 15 mV subthreshold depolarization of the soma propagated decrementally into the axon leading to a value of 9.43 mV at the presynaptic site (Figure 1C). This depolarization provoked the inactivation of axonal Kv1 channels leading to a broadening of the presynaptic spike, and the inactivation of Nav1.6 channels, producing a decrease in the presynaptic spike amplitude. Overall, the spike area was increased by 15.8%, leading to a 13.4% increase in spike-evoked calcium entry and a 36.8% increase in synaptic transmission (Figure 1C). Therefore, d-ADF amounted to 136.8% of the control amplitude at 134 μm from the soma in our model, which is similar to previously published d-ADF in pyramidal neurons (Shu et al., 2006; Kole et al., 2007; Bialowas et al., 2015).

The h-ADF is a much faster process that has been described in CA3 and L5 pyramidal neurons (Rama et al., 2015). A 200 ms hyperpolarization of the presynaptic neuron is enough to entail a recovery from inactivation of axonal Nav channels (Nav1.6 in pyramidal neurons), leading to an increase in axonal spike amplitude which produces an increase of spike-evoked Ca^2+^ entry and transmitter release (Figure 1B). Due to the slow recovery from inactivation of Kv1 channels, a 200 ms hyperpolarization has no effect on their availability. Therefore, h-ADF is a purely spike-amplitude dependent phenomenon (Rama et al., 2015). To verify the presence of h-ADF in the model, we injected current into the soma in order to produce a spike either generated from resting membrane potential (V_m_ = −70 mV) or after a 200 ms subthreshold hyperpolarization of the soma at −85 mV. Then, we measured the hyperpolarization value, the spike waveform, the spike-evoked-calcium entry and the postsynaptic response amplitude at the first presynaptic site (located 134 μm from the soma). We found that a 15 mV hyperpolarization of the soma propagated detrimentally into the axon leading to a value of 9.13 mV at the presynaptic site (Figure 1D). This hyperpolarization provoked the recovery from inactivation of axonal Nav1.6 channels, leading to a 17.8% increase in the presynaptic spike overshoot (without modification of its duration), an 11.9% increase in the spike-evoked Ca^2+^ entry and a 32.7% increase in synaptic transmission (Figure 1D). Therefore, h-ADF is similar to what has been obtained experimentally in pyramidal neurons (Rama et al., 2015).

We concluded that both d-ADF and h-ADF are reproduced by the model.

### d-ADF Spatial Extent Is Increased by Myelination

**Figure 2:**
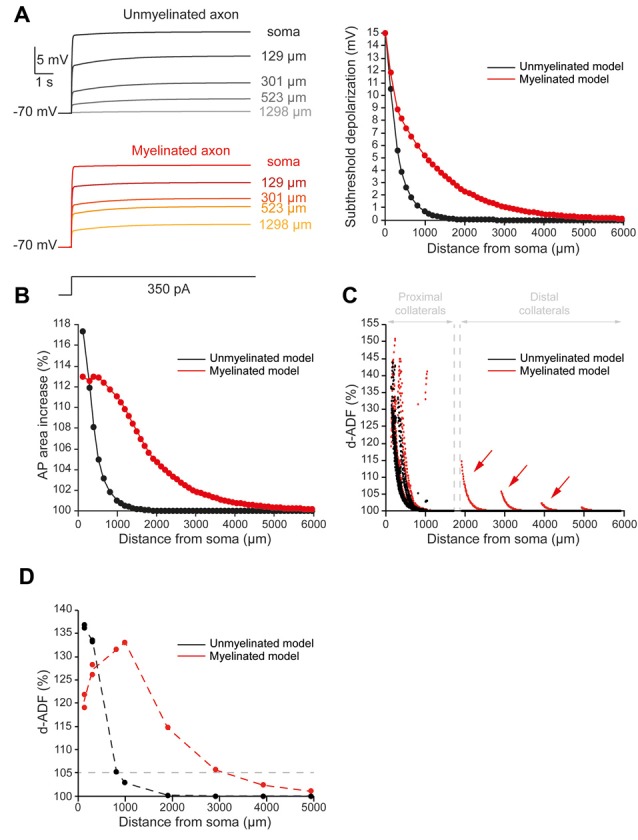
Axonal myelination increases d-ADF spatial extent. **(A)** Somatic subthreshold depolarization propagates farther in the myelinated axon than in the unmyelinated axon. Left, voltage traces showing the propagation of the somatic depolarization all along the axon (unmyelinated model: black to gray traces, myelinated model: red to yellow traces). Right, plot of the depolarization in the function of the distance from the soma in the unmyelinated (black) and the myelinated (red) model. **(B)** Plot of the depolarization-induced AP area increase in function of the distance from the soma in the unmyelinated (black) and the myelinated (red) model. **(C)** Plot of the depolarization-induced synaptic facilitation (d-ADF) in the function of the distance from the soma in the 1,982 presynaptic sites located in axon collaterals (black dots: unmyelinated model, red dots: myelinated model). Note that d-ADF is present in presynaptic sites located into distant collaterals only in the myelinated model (red arrows). **(D)** Plot of d-ADF measured in the first presynaptic site of each collateral in the function of the distance from the soma (black: unmyelinated model, red: myelinated model).

To quantify the spatial extent of d-ADF, we measured the subthreshold depolarization and the AP area all along the main axon. We found that the depolarization propagated with a space constant of 303.4 μm into the main axon, close to previously published values in L5 pyramidal neurons (Shu et al., 2006; Kole et al., 2007; Figure 2A, black trace). This led to a space constant for depolarization-induced AP area increase of 338.4 μm into the main axon (Figure 2B, black trace). Then, in order to observe d-ADF, we computed synaptic transmission in the 1982 presynaptic sites located in axon collaterals (Figure 2C, black trace). In this configuration, we found that 31.4% of the presynaptic sites presented a d-ADF of at least 5% (Figure 2C, black dots). These presynaptic sites were all located in the proximal collaterals and were on average 307 ± 94.5 μm away from the soma. In order to determine the spatial extent of d-ADF in the model, we plotted the value of d-ADF measured at the first presynaptic site of each collateral as a function of the distance from the soma (Figure 2D, black trace). We defined the spatial extent of d-ADF as the point where this curve crossed the value of 105% for d-ADF. We found that the spatial extent of d-ADF was 836 μm (Figure 2D, black trace). Therefore, d-ADF in an unmyelinated axon is a local phenomenon restricted to proximal synapses (i.e., corresponding to an axonal path smaller than 1 mm; Sasaki et al., 2012).

To evaluate the impact of myelination on the d-ADF spatial extent, we simulated myelinated internodes in the main axon while we left the collaterals unmyelinated. In this model, the main axon was myelinated except at the branching points of the collaterals where 1.5 μm-long nodes of Ranvier were simulated. However, if the distance between two branching points was longer than 200 μm, this part of the main axon has been divided into 100 μm-long internodes with nodes of Ranvier in between. Therefore, the myelinated internodes present a length that varies from 99 to 180 μm (mean value of 101.1 μm), which corresponds to previously published values in L5 pyramidal neurons (Arancibia-Cárcamo et al., 2017). To simulate myelinated internodes, we removed the voltage-gated conductance (Nav1.6 and Kv1) from these compartments, and we added a layer of myelin sheath which conductance and capacitance correspond to 15 myelin wraps (see “Materials and Methods” section and Supplementary Figure S3 for myelin modeling). In that case, the myelin sheath conductance was equal to the axonal leak channels conductance divided by a factor 30 and the myelin sheath capacitance was equal to the axonal capacitance divided by 30 (see “Materials and Methods” section and Table 2: Myelinated axon 1). These values of myelin membrane conductance and capacitance corresponds to what could be expected for a myelin sheath constituted of 15 myelin wraps, a value that we deduced from a g-ratio of 0.698 found in L5 pyramidal neurons (Cohen et al., 2020), the diameter of the main axon in the reconstructed neuron (1.14 μm) and a periaxonal space of 12.3 nm between the internode’s plasma membrane and the myelin sheath (Cohen et al., 2020; see “Materials and Methods” section for myelin wraps modeling). Finally, the periaxonal space resistivity was set to 53.7 Ωcm (Cohen et al., 2020). The myelinated internodes are separated by nodes of Ranvier. We simulated nodes of Ranvier by increasing voltage-gated sodium and potassium conductance in order to ensure the spike propagation all along the axon (Table 2: Myelinated axon 1). Importantly, the exact densities of Nav1.6 and Kv into the nodes of Ranvier were determined in order to preserve the spike waveform at resting membrane potential in the middle of the main axon (10 mm from the soma; Supplementary Figure S2). Finally, the density of the leak channels in the nodes of Ranvier was the same as in the non-myelinated axon.

As expected, we found that myelination increased the velocity of the AP conduction (Supplementary Figure S4). More importantly, we found that the subthreshold depolarization of the soma propagated into the myelinated main axon with a space constant of 906.6 μm (Figure 2A, red trace). Therefore, the myelination entailed a 2.99-fold increase in axonal length constant in our model. In consequence, the myelination increased the spatial extent of the depolarization-induced AP area enhancement (Figure 2B, red trace). Importantly, it was not possible to extract a space constant for the depolarization-induced AP area enhancement because it did not present a monotonic decay in the myelinated axon (see “Materials and Methods” section for space constant calculation). In fact, this parameter evolved in a biphasic manner along the axon: it was stable during the first 650 micrometers then it decreased with the distance from the soma (Figure 2B, red trace). This is easily understandable if one takes into account the two opposite effects of the subthreshold depolarization on the AP shape: it increases its duration and reduces its amplitude. The interplay between these two effects creates the biphasic behavior of the AP area increase in our myelinated model. However, despite the impossibility to calculate a space constantly, our model showed that myelination led to a major increase in the axonal portion affected by the depolarization-induced increase in the AP area. Consequently, we found an increase in the presynaptic sites proportion that presents at least 5% d-ADF (36% instead of 31.4%; Figure 2C, red dots). These presynaptic sites were located both on proximal and distal collaterals and were on average 382.7 ± 318.2 μm away from the soma. Importantly, we found that some presynaptic sites presented d-ADF while they were located more than 2,000 μm away from the soma (Figure 2C, red dots). In fact, the spatial extent of d-ADF was found to be 3,124 μm in this configuration (Figure 2D, red trace). Therefore, the myelination of the main axon allowed the propagation of the somatic subthreshold depolarization to distant collaterals and entailed a major increase in the d-ADF spatial extent.

### h-ADF Spatial Extent Is Increased by Myelination

**Figure 3:**
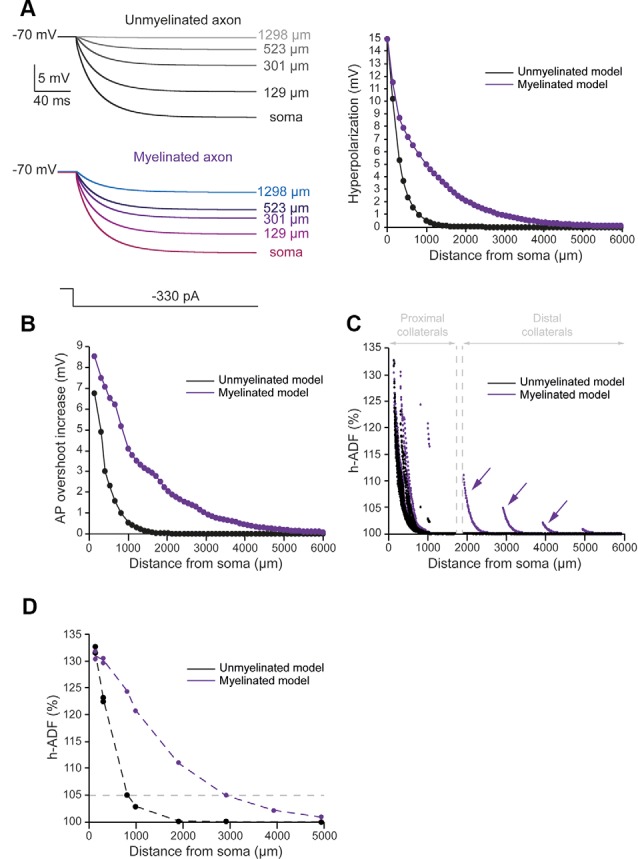
Axonal myelination increases h-ADF spatial extent. **(A)** Somatic subthreshold hyperpolarization propagates farther in the myelinated axon than in the unmyelinated axon. Left, voltage traces showing the propagation of the somatic hyperpolarization all along the axon (unmyelinated model: black to gray traces, myelinated model: purple to blue traces). Right, plot of the hyperpolarization in the function of the distance from the soma in the unmyelinated (black) and the myelinated (purple) model. **(B)** Plot of the hyperpolarization-induced AP overshoot increase in function of the distance from the soma in the unmyelinated (black) and the myelinated (purple) model. **(C)** Plot of the hyperpolarization-induced synaptic facilitation (h-ADF) in the function of the distance from the soma in the 1,982 presynaptic sites located in axon collaterals (black dots: unmyelinated model, red dots: myelinated model). Note that h-ADF is present in presynaptic sites located into distant collaterals only in the myelinated model (purple arrows). **(D)** Plot of h-ADF measured in the first presynaptic site of each collateral in the function of the distance from the soma (black: unmyelinated model, purple: myelinated model).

To evaluate the spatial extent of h-ADF, we measured the hyperpolarization and AP overshoot all along the main axon. We also measured the synaptic transmission at presynaptic sites located in axon collaterals. In the unmyelinated model, we found that the hyperpolarization propagated with a space constant of 294.2 μm (Figure 3A, black trace). This leads to a space constant for an AP overshoot increase of 360.3 μm (Figure 3B, black trace). 29.6% of the presynaptic sites displayed an h-ADF of at least 5% (Figure 3C, black dots). These presynaptic sites were all located in the proximal collaterals and were on average 300.3 ± 91.8 μm away from the soma (Figure 3C, black dots). The h-ADF spatial extent was found to be 815 μm (Figure 3D, black trace).

In the myelinated model, the hyperpolarization propagated into the main axon with a space constant of 858.5 μm (Figure 3A, purple trace). Therefore, the space constant of the propagation of subthreshold hyperpolarization is increased by a factor 2.92 compared to an unmyelinated main axon. This leads to an augmentation of the space constant of hyperpolarization-induced AP overshoot increase (Figure 3B, purple trace). Consequently, we found an increase in the proportion of presynaptic sites that displayed at least 5% h-ADF (from 29.6 to 34%; Figure 3C, purple dots). These presynaptic sites were located both on proximal and distal collaterals and were on average 365.5 μm ± 281.24 μm away from the soma. Importantly, as for d-ADF, we found that some presynaptic sites still present h-ADF while they were located more than 2,000 μm away from the soma (Figure 3C, purple dots). In fact, we found that the h-ADF spatial extent was 2,894 μm in the myelinated model (Figure 3D, purple trace). Therefore, similar to its effect on d-ADF, the myelination induced a major increase in the h-ADF spatial extent.

### The Enhanced Spatial Extent of ADFs Is Not Due to Hot Spots of Ion Channels at Nodes of Ranvier

One major difficulty of our approach is the fact that Nav1.6 and Kv1 axonal densities were different in the myelinated and the unmyelinated model (Table 2). Therefore, the increase in ADFs spatial extent in the myelinated axon could arise either from the presence of myelin sheaths at the internodes or from the increase in ion channels densities into nodes of Ranvier. To determine the key parameter controlling the increase in ADF spatial extent, we performed a simulation with a hybrid main axon presenting the same parameters values than the unmyelinated main axon except at specific hot spots located at the same location than nodes of Ranvier of the myelinated model (Table 2: Hybrid axon). At these hot spots, the parameters values are identical to those of the nodes of Ranvier of the myelinated model. Therefore, this main axon is a hybrid model that presents nodes of Ranvier-like hot spots separated by portions of non-myelinated axon.

In this hybrid model, we found that the subthreshold depolarization propagated into the main axon with a space constant of 301.6 μm, close to the value found in the unmyelinated main axon (303.4). The depolarization-induced enhancement of the AP area propagated into the main axon with a space constant of 327 μm, close to the value found in the unmyelinated main axon (338.4 μm). We found that 31.2% of the presynaptic sites presented a d-ADF of at least 5% and that they were located on average 306.1 ± 93.9 μm from the soma. These values are close to the values we found for the unmyelinated model (31.4% and 307 ± 94.5 μm). Moreover, d-ADF has a spatial extent of 816 μm in the hybrid axon, close to the value of 836 μm found in the unmyelinated axon. Concerning h-ADF, we found that the hyperpolarization propagated into the main axon with a space constant of 293.4 μm which is close to the value found in the unmyelinated main axon (294.2). The hyperpolarization-induced increase in AP overshoot propagated into the main axon with a space constant of 349 μm, close to the value found in the unmyelinated main axon (360.3 μm). We found that 29.6% of the presynaptic sites presented an h-ADF of at least 5% and that they were located on average 299.5 ± 89.3 μm from the soma. These values are close to the values we found for the unmyelinated model (29.6% and 300.3 ± 91.8 μm). Additionally, h-ADF presented a spatial extent of 808 μm in the hybrid axon, close to the value found in the unmyelinated model (814 μm). Therefore, the spatial extents of d-ADF and h-ADF were similar in the non-myelinated model and in the hybrid model. We conclude that the increase in the ADFs spatial extents in the myelinated model was due to the presence of myelinated internodes and was not an artifact due to high Nav1.6 and Kv1 densities in the nodes of Ranvier.

### Effect of Myelination Parameters on ADFs Spatial Extent

**Figure 4:**
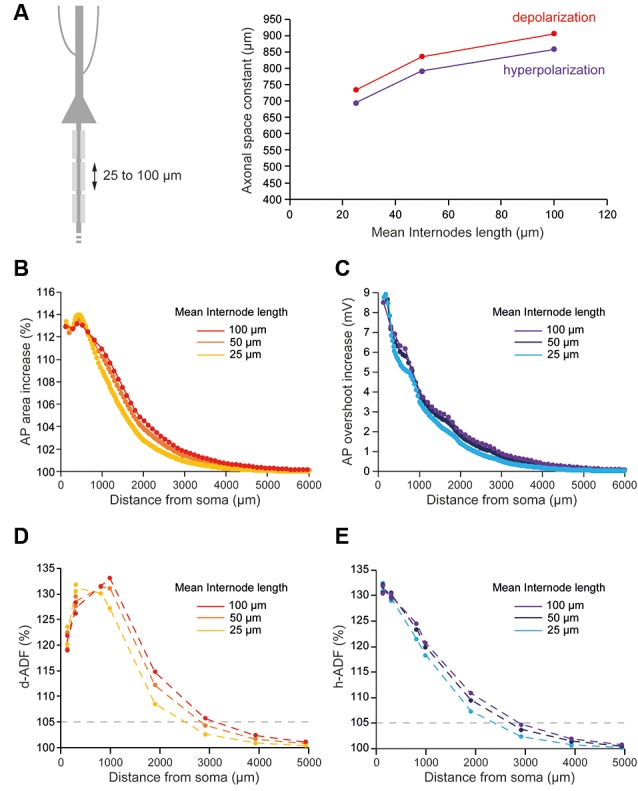
Internodal length mildly determines ADFs spatial extent. **(A)** The reduction of the internodal length leads to a decreased axonal length constant. Left, schematic representation of the model with varying average internodal lengths. Right, plot of the axonal length constant for a 10 s somatic depolarization (red) or a 200 ms somatic hyperpolarization (purple) in the function of the internodal length. **(B)** Plot of the depolarization-induced AP area increase in function of the distance from the soma for the different average internodal lengths. **(C)** Plot of the hyperpolarization-induced AP overshoot increase in function of the distance from the soma for the different average internodal lengths. **(D)** Plot of d-ADF measured in the first presynaptic site of each collateral for the different internodal lengths. **(E)** Plot of h-ADF measured in the first presynaptic site of each collateral for the different internodal lengths.

Several studies showed that the length of the internodes is variable during development and among neuronal types, which may impact the axonal length constant. In L5 pyramidal neurons, they have been shown to vary between 30 and 150 μm from cell to cell (Arancibia-Cárcamo et al., 2017). In order to observe the effects of internodal length modifications on ADFs spatial extents, we divided all the internodes lengths by 2 (average internodal length: 50.59 μm) or 4 (average internodal length: 25.35 μm) while keeping the distance of collaterals from the soma constant (Figure 4A). For each condition (internodal length divided by 2 or 4), the axonal densities of Nav1.6 and Kv1 channels were modified in order to keep the axonal spike waveform at the resting membrane potential similar to the one in the non-myelinated axon (Table 2: Myelinated axon 1, 2, 3; Supplementary Figure S2). Moreover, E_leak_ was slightly modified to maintain the resting membrane potential at −70 mV in the axon (Table 2: Myelinated axon 1, 2, 3). We found a mild decrease in the propagation of subthreshold depolarization with the internodal length reduction (Figure 4A, red trace). Consequently, the internodal length reduction provoked a small decrease in the space constant for depolarization-induced enhancement of the AP area (Figure 4B). This led to a limited diminution of the d-ADF spatial extent with internodes shortening (Figure 4D). In fact, d-ADF spatial extent was found to be 2,501 μm, 2,830 μm and 3,124 μm for average internodal lengths of respectively 25.35 μm, 50.59 μm and 101.1 μm (Figure 4D). Similarly, we found a decrease in the axonal length constant for hyperpolarization when the internodal length was reduced (Figure 4A, purple trace). As a result, hyperpolarization-induced AP overshoot enhancement displayed a shorter space constant (Figure 4C) leading to a decrease in h-ADF spatial extent (Figure 4E). We concluded that internodal length mildly influences ADFs spatial extents in our model.

**Figure 5:**
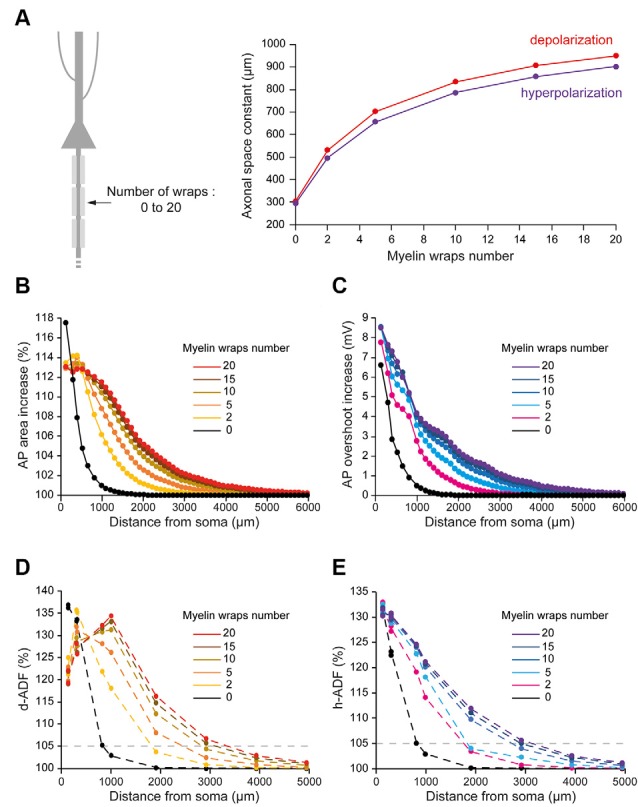
Number of myelin wraps determines ADF spatial extent. **(A)** Increasing the myelin wraps number leads to an increased axonal length constant. Left, schematic representation of the model with varying myelin wraps number. Right, Plot of the axonal length constant for a 10 s somatic depolarization (red) or a 200 ms somatic hyperpolarization (purple) in the function of myelin wraps number. **(B)** Plot of the depolarization-induced AP area increase in function of the distance from the soma for the different myelin wraps numbers. **(C)** Plot of the hyperpolarization-induced AP overshoot increase in function of the distance from the soma for the different myelin wraps numbers. **(D)** Plot of d-ADF measured in the first presynaptic site of each collateral for the different myelin wraps numbers. **(E)** Plot of h-ADF measured in the first presynaptic site of each collateral for the different myelin wraps numbers.

The number of myelin wraps increases during development (Looney and Elberger, 1986; Battefeld et al., 2019). Moreover, the number of myelin wraps has been found to vary between 5 and 20 in mature L5 pyramidal neurons (Cohen et al., 2020). We, therefore, explored the effect of myelin wraps number on d- and h-ADF spatial extents. For this, we compared the spatial extents of ADFs into axons which internodes are ensheathed by 0, 2, 5, 10, 15 or 20 myelin wraps (Figure 5A; see “Materials and Methods” section for the modeling of myelin wraps number). We had to modify the densities of Nav1.6 and Kv1 channels in the axon in order to keep the axonal spike waveform and the E_leak_ to maintain the resting membrane potential for each myelin wraps number (Table 2 and Supplementary Figure S2: Non-myelinated axon and Myelinated axon 1, 4, 5, 6, 7). We found that the subthreshold depolarization space constant for an axon which internodes are ensheathed in 0 myelin wraps was 303.4 μm while it was 531.2 μm, 702.2 μm, 834.3 μm, 906.6 μm and 949.8 μm for axons which internodes are ensheathed respectively into 2, 5, 10, 15 and 20 myelin wraps (Figure 5A, red trace). The depolarization space constant followed the exponential relationship sp = 313.24 + 636.2 * (1 − e^−0.189*N^) where sp is the depolarization space constant and N the number of myelin wraps. Consequently, the spatial extent of both depolarization-induced AP area enhancement and d-ADF greatly increased with the number of myelin wraps ensheathing internodes (Figures 5B,D). In fact, the spatial extent of d-ADF was found to be 836 μm, 1,827 μm, 2,453 μm, 2,840 μm, 3,124 μm and 3,351 μm when the internodes were ensheathed into respectively 0, 2, 5, 10, 15 or 20 myelin wraps. Similarly, we found that the subthreshold hyperpolarization space constant greatly increased with the number of myelin wraps following the relationship sp = 303.02 + 604.3* (1 − e^−0.175*N^) (Figure 5A). Therefore, increasing the number of myelin wraps led to an increase in the spatial extent of both hyperpolarization-induced AP overshoot enhancement and h-ADF (Figures 5C,E). We conclude that the number of myelin wraps ensheathing the internodes is a major determinant of both d-ADF and h-ADF spatial extents, suggesting that in the young adult rat ADFs may extend to more than 2 mm away from the soma.

## Discussion

In this study, we show using a computational approach that the axonal myelination may expand both the axonal length constant as well as d- and h-ADF spatial extents by around a factor 3. Furthermore, we show that ADFs spatial extents are critically determined by the number of myelin wraps ensheathing the axon and more modestly by the internodal length. Our work, therefore, suggests that myelinated projection paths such as cortico-striatal or cortico-collicular pathways with an axonal distance ranging between 2 and 3 mm may well express d- and h-ADF.

### Axonal Length Constant

Axonal length constant depends on several geometrical and electrical parameters as the axonal diameter, the presence of myelin, the number of branch-points or the duration of the voltage shift imposed in the soma. A length constant of ~2 mm has been estimated in myelinated axons of motoneurons (Gogan et al., 1983). In hippocampal axons, the length constant is inversely proportional to the number of branch-points (Sasaki et al., 2012). In L5 pyramidal neurons, length constant is 120 μm for a 10 ms depolarization whereas it can reach 1,000 μm for a 200 ms depolarization (Christie and Jahr, 2009), showing that the propagation of voltage along the axon depends on the frequency of the signal imposed in the somatic compartment.

In L5 pyramidal neurons, the value of the main axon length constant has been estimated between 417 and 1,180 μm for a long subthreshold depolarization (0.2–10 s; Kole et al., 2007; Shu et al., 2007a; Christie and Jahr, 2009; Cohen et al., 2020). Based on our modeling, we propose that this large range of reported values is due to the variability of the axonal myelination in the different studies. In fact, the myelination increases the axonal length constant in our model (Figures 2A,B). Importantly, the study from Shu and colleagues was performed on ferrets, a species in which L5 pyramidal neurons myelination starts distally (<350 μm from the soma; Shu et al., 2007a). This could explain the smaller axonal length constant found in this study (417 μm) compared to other studies performed on rats (553–1,180 μm; Kole et al., 2007; Christie and Jahr, 2009; Cohen et al., 2020) in which the axonal myelination starts just after the axonal initial segment (Battefeld et al., 2019). Moreover, we propose that the large range of axonal length constants found in rat L5 pyramidal neurons is due to the variability of myelination between neurons. In fact, the number of myelin wraps can vary between 5 and 20 in mature L5 pyramidal neurons (Cohen et al., 2020). In our model, the axonal length constant increases with the number of myelin wrap leading to a value of 531 μm for 5 myelin wraps and a value of 949.8 μm for 20 myelin wraps (Figure 5A). However, while we found a 3-fold effect of myelination on axonal length constant in our simulations, this still needs to be experimentally explored by performing recordings of L5 pyramidal neurons treated by demyelinating drugs such as cuprizone (Hamada et al., 2017).

Importantly, axonal length constant also depends on the axonal membrane resistance. One must consider that somatodendritic membrane resistance decreases during the development (Atkinson and Williams, 2009) which leads to a decrease in the dendritic length constant. If this phenomenon also occurs in axons, we may have overestimated the axonal length constant of mature L5 pyramidal neurons. Nevertheless, the value of 0.333 pS.μm^−2^ for leak channel density used in our model corresponds to a membrane resistance of 30.03 kΩ.cm^2^ which is close to the value of 24.6 kΩ.cm^2^ recently estimated by Cohen and colleagues in mature L5 pyramidal neurons (Cohen et al., 2020). Moreover, some studies suggest that the axonal membrane resistance may be much lower than dendritic membrane resistance leading to a large axonal length constant (Dover et al., 2016).

### ADF Spatial Extent

The axonal length constant and ADFs spatial extents are biophysically related as ADFs tightly depend on the propagation of somatic subthreshold voltage shifts into the axon leading to the modulation of AP parameters in the presynaptic bouton. The space constant of the depolarization-induced AP broadening underlying d-ADF has been estimated to be near 675 μm in L5 pyramidal cell axons (Kole et al., 2007). In our study, we found that the space constant of the depolarization-induced AP broadening is approximately 338.4 μm for the unmyelinated model but was greatly enhanced in the myelinated model (Figure 2B). In fact, we showed that increasing the length of internodes or the number of myelin wraps, which occurs during the development of L5 pyramidal neurons (Battefeld et al., 2019), leads to an increase in axonal length constant and ADFs spatial extent (Figures 4, 5).

### Myelination Parameters

What is the number of myelin wraps in axons? Myelin wraps in peripheral axons linearly depend on the axon diameter and may vary between 10 and 160 (Arbuthnott et al., 1980; Berthold and Carlstedt, 1982). In the CNS, the number of myelin wraps has been estimated to vary between 5 and 20 wraps (Looney and Elberger, 1986; Bakiri et al., 2011; Harris and Attwell, 2012; Snaidero et al., 2014; Arancibia-Cárcamo et al., 2017; Cohen et al., 2020). Our results suggest that the number of myelin wraps has a critical impact on both the axonal length constant and the ADFs spatial extents (Figure 5). In fact, the space constant for subthreshold depolarization and hyperpolarization went from ~500 μm with 2 myelin wraps to ~900 μm for 20 myelin wraps in our model (Figure 5A). As a consequence, the spatial extent for d- and h-ADF was found to increase in proportion (Figures 5D,E). Therefore, we suggest that the variability of the myelin wraps number found in mature L5 pyramidal neurons could entail a variability of ADFs spatial extent in this cell type. During development, the number of myelin lamellae increases from 0 before birth to maximal levels before sexual maturity (Berthold and Carlstedt, 1982). The myelin thickness also depends on electrical activity (Kaller et al., 2017; Suminaite et al., 2019). Therefore, our model suggests that ADFs spatial extent may increase during the development and vary as a function of neuronal electrical activity.

Regarding the internodal length, we found that this parameter, known to critically determine the conduction velocity (Rushton, 1951; Wu et al., 2012), mildly influenced the axonal length constant and ADFs spatial extents in our model (Figure 4).

### Extended ADF in Myelinated Axonal Paths *in situ*?

Due to a large amount of missing data about axonal physiology, our model presents several unconstrained parameters such as the exact axonal channels density and nature at nodes of Ranvier and internodes or the precise axonal membrane resistance. While it seems likely that the electrical isolation provided by the myelination increases the axonal length constant and ADFs spatial extents, experiments are needed to assess if ADFs can occur at distal synaptic connections (>2 mm). In fact, d-ADF and h-ADF have been mostly studied either in unmyelinated axons or in immature brain circuits in which the myelin is weakly present during early development. As a consequence, the presence of ADFs has never been evaluated in fully myelinated axonal paths. Our data suggest that short projection paths as thalamocortical projection may well express d- and h-ADF. Indeed, in this structure myelination starts rapidly during postnatal development, the length of thalamic axons (~2 mm) is compatible with ADF and paired-recording from thalamocortical neurons can be obtained *in vitro* (Hu and Agmon, 2016) and *in vivo* (Bruno and Sakmann, 2006). Further experimental work will probably help answer this question.

## Data Availability Statement

The datasets generated for this study are available on request to the corresponding author.

## Authors Contributions

MZ and DD designed the article and wrote the manuscript. MZ performed the simulations and built the figures.

## Conflict of Interest

The authors declare that the research was conducted in the absence of any commercial or financial relationships that could be construed as a potential conflict of interest.

## Supplementary Material

FIGURE S1 Model morphology. Representation of the model morphology based on the reconstructed neuron NMO_07763. Note the axonal portion that has been added to the reconstructed neuron to extend the axonal tree.

FIGURE S2 Axonal spike waveform at the resting membrane potential is similar in the different simulations. **(A)** Schematic representation of the different models of the study. **(B)** Spike waveform at the resting membrane potential (−70 mV) recorded in the middle of the axon (10 mm from the soma) in the different models. Note the similarity of basal axonal spike waveform in the different models.

FIGURE S3 Myelin sheath modeling. Up, schematic representation of two internodes and one node of Ranvier. Down, equivalent electric circuit used in the model. Gmy, myelin sheath conductance; Cmy, myelin sheath capacitance; Rp, periaxonal space axial resistance; Gax, axonal passive conductance; Cax, axonal capacitance; Ra, intra-axonal axial resistance; Kv1, Kv1 channels conductance; Nav, axonal Nav channels conductance (Nav1.6 channels conductance).

FIGURE S4 Myelination increase velocity of spike propagation into the main axon. Left, voltage traces showing AP propagation along the main axon in the unmyelinated and the myelinated model (Table 2: Myelinated axon 1). Right, plot of the AP latency in the function of the distance from the soma in the myelinated and the unmyelinated model.

## References

[B1] Alcami P. El Hady A. (2019). Axonal computations. Front. Cell. Neurosci. 13:413. 10.3389/fncel.2019.00413 31619963 PMC6759653

[B2] Alle H. Geiger J. R. (2006). Combined analog and action potential coding in hippocampal mossy fibers. Science 311, 1290–1293. 10.1126/science.1119055 16513983

[B3] Arancibia-Cárcamo I. L. Ford M. C. Cossell L. Ishida K. Tohyama K. Attwell D. (2017). Node of Ranvier length as a potential regulator of myelinated axon conduction speed. Elife 6:e23329. 10.7554/eLife.23329 28130923 PMC5313058

[B4] Arbuthnott E. R. Boyd I. A. Kalu K. U. (1980). Ultrastructural dimensions of myelinated peripheral nerve fibres in the cat and their relation to conduction velocity. J. Physiol. 308, 125–157. 10.1113/jphysiol.1980.sp013465 7230012 PMC1274542

[B5] Ascoli G. A. Donohue D. E. Halavi M. (2007). NeuroMorpho.Org: a central resource for neuronal morphologies. J. Neurosci. 27, 9247–9251. 10.1523/JNEUROSCI.2055-07.2007 17728438 PMC6673130

[B6] Atkinson S. E. Williams S. R. (2009). Postnatal development of dendritic synaptic integration in rat neocortical pyramidal neurons. J. Neurophysiol. 102, 735–751. 10.1152/jn.00083.2009 19458150 PMC2724330

[B7] Bakiri Y. Káradóttir R. Cossell L. Attwell D. (2011). Morphological and electrical properties of oligodendrocytes in the white matter of the corpus callosum and cerebellum. J. Physiol. 589, 559–573. 10.1113/jphysiol.2010.201376 21098009 PMC3055543

[B8] Battefeld A. Popovic M. A. de Vries S. I. Kole M. H. P. (2019). High-frequency microdomain Ca^2+^ transients and waves during early myelin internode remodeling. Cell Rep. 26, 182.e5–191.e5. 10.1016/j.celrep.2018.12.039 30605675 PMC6316190

[B9] Berthold C. H. Carlstedt T. (1982). Myelination of S1 dorsal root axons in the cat. J. Comp. Neurol. 209, 225–232. 10.1002/cne.902090302 7130453

[B10] Bialowas A. Rama S. Zbili M. Marra V. Fronzaroli-Molinieres L. Ankri N.. (2015). Analog modulation of spike-evoked transmission in CA3 circuits is determined by axonal Kv1.1 channels in a time-dependent manner. Eur. J. Neurosci. 41, 293–304. 10.1111/ejn.12787 25394682

[B11] Bischofberger J. Geiger J. R. Jonas P. (2002). Timing and efficacy of Ca^2+^ channel activation in hippocampal mossy fiber boutons. J. Neurosci. 22, 10593–10602. 10.1523/JNEUROSCI.22-24-10593.2002 12486151 PMC6758411

[B12] Bruno R. M. Sakmann B. (2006). Cortex is driven by weak but synchronously active thalamocortical synapses. Science 312, 1622–1627. 10.1126/science.1124593 16778049

[B13] Castelfranco A. M. Hartline D. K. (2015). The evolution of vertebrate and invertebrate myelin: a theoretical computational study. J. Comput. Neurosci. 38, 521–538. 10.1007/s10827-015-0552-x 25832903

[B14] Christie J. M. Jahr C. E. (2009). Selective expression of ligand-gated ion channels in L5 pyramidal cell axons. J. Neurosci. 29, 11441–11450. 10.1523/JNEUROSCI.2387-09.2009 19759293 PMC2814532

[B15] Cohen C. C. H. Popovic M. A. Klooster J. Weil M. T. Mobius W. Nave K. A.. (2020). Saltatory conduction along myelinated axons involves a periaxonal nanocircuit. Cell 180, 311–322.e15. 10.1016/j.cell.2019.11.039 31883793 PMC6978798

[B16] Debanne D. Bialowas A. Rama S. (2013). What are the mechanisms for analogue and digital signalling in the brain? Nat. Rev. Neurosci. 14, 63–69. 10.1038/nrn3361 23187813

[B17] Dover K. Marra C. Solinas S. Popovic M. Subramaniyam S. Zecevic D.. (2016). FHF-independent conduction of action potentials along the leak-resistant cerebellar granule cell axon. Nat. Commun. 7:12895. 10.1038/ncomms12895 27666389 PMC5052690

[B18] Gogan P. Gueritaud J. P. Tyc-Dumont S. (1983). Comparison of antidromic and orthodromic action potentials of identified motor axons in the cat’s brain stem. J. Physiol. 335, 205–220. 10.1113/jphysiol.1983.sp014529 6875874 PMC1197348

[B19] Hamada M. S. Popovic M. A. Kole M. H. (2017). Loss of saltation and presynaptic action potential failure in demyelinated axons. Front. Cell. Neurosci. 11:45. 10.3389/fncel.2017.00045 28289377 PMC5326753

[B20] Harris J. J. Attwell D. (2012). The energetics of CNS white matter. J. Neurosci. 32, 356–371. 10.1523/JNEUROSCI.3430-11.2012 22219296 PMC3272449

[B21] Hay E. Hill S. Schürmann F. Markram H. Segev I. (2011). Models of neocortical layer 5b pyramidal cells capturing a wide range of dendritic and perisomatic active properties. PLoS Comput. Biol. 7:e1002107. 10.1371/journal.pcbi.1002107 21829333 PMC3145650

[B22] Hu H. Agmon A. (2016). Differential excitation of distally versus proximally targeting cortical interneurons by unitary thalamocortical bursts. J. Neurosci. 36, 6906–6916. 10.1523/JNEUROSCI.0739-16.2016 27358449 PMC4926238

[B23] Hu W. Tian C. Li T. Yang M. Hou H. Shu Y. (2009). Distinct contributions of Na_v_1.6 and Na_v_1.2 in action potential initiation and backpropagation. Nat. Neurosci. 12, 996–1002. 10.1038/nn.2359 19633666

[B24] Kaller M. S. Lazari A. Blanco-Duque C. Sampaio-Baptista C. Johansen-Berg H. (2017). Myelin plasticity and behaviour-connecting the dots. Curr. Opin. Neurobiol. 47, 86–92. 10.1016/j.conb.2017.09.014 29054040 PMC5844949

[B25] Kole M. H. Letzkus J. J. Stuart G. J. (2007). Axon initial segment Kv1 channels control axonal action potential waveform and synaptic efficacy. Neuron 55, 633–647. 10.1016/j.neuron.2007.07.031 17698015

[B26] Looney G. A. Elberger A. J. (1986). Myelination of the corpus callosum in the cat: time course, topography, and functional implications. J. Comp. Neurol. 248, 336–347. 10.1002/cne.902480304 3722461

[B27] Rama S. Zbili M. Bialowas A. Fronzaroli-Molinieres L. Ankri N. Carlier E.. (2015). Presynaptic hyperpolarization induces a fast analogue modulation of spike-evoked transmission mediated by axonal sodium channels. Nat. Commun. 6:10163. 10.1038/ncomms10163 26657943 PMC4682119

[B28] Romand S. Wang Y. Toledo-Rodriguez M. Markram H. (2011). Morphological development of thick-tufted layer V pyramidal cells in the rat somatosensory cortex. Front. Neuroanat. 5:5. 10.3389/fnana.2011.00005 21369363 PMC3043270

[B29] Rushton W. A. (1951). A theory of the effects of fibre size in medullated nerve. J. Physiol. 115, 101–122. 10.1113/jphysiol.1951.sp004655 14889433 PMC1392008

[B30] Sasaki T. Matsuki N. Ikegaya Y. (2012). Effects of axonal topology on the somatic modulation of synaptic outputs. J. Neurosci. 32, 2868–2876. 10.1523/JNEUROSCI.5365-11.2012 22357869 PMC6621900

[B31] Scott R. Ruiz A. Henneberger C. Kullmann D. M. Rusakov D. A. (2008). Analog modulation of mossy fiber transmission is uncoupled from changes in presynaptic Ca^2+^. J. Neurosci. 28, 7765–7773. 10.1523/JNEUROSCI.1296-08.2008 18667608 PMC2685171

[B32] Shu Y. Duque A. Yu Y. Haider B. McCormick D. A. (2007a). Properties of action-potential initiation in neocortical pyramidal cells: evidence from whole cell axon recordings. J. Neurophysiol. 97, 746–760. 10.1152/jn.00922.2006 17093120

[B34] Shu Y. Yu Y. Yang J. McCormick D. A. (2007b). Selective control of cortical axonal spikes by a slowly inactivating K^+^ current. Proc. Natl. Acad. Sci. U S A 104, 11453–11458. 10.1073/pnas.0702041104 17581873 PMC2040919

[B33] Shu Y. Hasenstaub A. Duque A. Yu Y. McCormick D. A. (2006). Modulation of intracortical synaptic potentials by presynaptic somatic membrane potential. Nature 441, 761–765. 10.1038/nature04720 16625207

[B35] Snaidero N. Möbius W. Czopka T. Hekking L. H. Mathisen C. Verkleij D.. (2014). Myelin membrane wrapping of CNS axons by PI(3,4,5)P3-dependent polarized growth at the inner tongue. Cell 156, 277–290. 10.1016/j.cell.2013.11.044 24439382 PMC4862569

[B36] Suminaite D. Lyons D. A. Livesey M. R. (2019). Myelinated axon physiology and regulation of neural circuit function. Glia 67, 2050–2062. 10.1002/glia.23665 31233642 PMC6772175

[B37] Wu L. M. Williams A. Delaney A. Sherman D. L. Brophy P. J. (2012). Increasing internodal distance in myelinated nerves accelerates nerve conduction to a flat maximum. Curr. Biol. 22, 1957–1961. 10.1016/j.cub.2012.08.025 23022068 PMC3482659

[B38] Zbili M. Debanne D. (2019). Past and future of analog-digital modulation of synaptic transmission. Front. Cell. Neurosci. 13:160. 10.3389/fncel.2019.00160 31105529 PMC6492051

