# Corrigendum: Myelination Increases the Spatial Extent of Analog-Digital Modulation of Synaptic Transmission: A Modeling Study

**DOI:** 10.3389/fncel.2020.00099

**Published:** 2020-05-04

**Authors:** Mickaël Zbili, Dominique Debanne

**Affiliations:** ^1^Lyon Neuroscience Research Center, INSERM U1028-CNRS UMR5292-Université Claude Bernard Lyon1, Lyon, France; ^2^UNIS UMR 1072 INSERM, AMU, Marseille, France

**Keywords:** myelin, axon, axonal space constant, analog digital facilitation, spike shape, ion channels, axonal length constant

In the original article, there was an error in the equation *W* = *A* * *Q*_*Ca*_^2+^ describing how we computed the synaptic strength from the calcium charge in the presynaptic terminals. Actually, we used the following equation in the model: *W* = *A* * (*Q*_*Ca*_^2+^)^2.5^. In consequence, a correction has been made to the Materials and Methods section, subsection Postsynaptic Responses, first paragraph:

“To obtain the postsynaptic responses, we used Alpha Synapse Point Processes from Neuron 7.6 inserted into postsynaptic cells. The weights of the synapses were calculated using the charge of the spike-evoked Ca^2+^ entry in the presynaptic sites with the following power law:

$$W=A*(Q_{Ca}{}^{2+}{)}^{2.5}$$

where W is the synaptic weight, A is a scaling factor and Q_*Ca*_ is the charge of the spike-evoked Ca^2+^ current (Scott et al., 2008). Therefore, an increase in the Ca^2+^ entry produced by an increase in presynaptic spike amplitude or duration led to an increase in the postsynaptic response amplitude.”

The authors apologize for this error and state that this does not change the scientific conclusions of the article in any way. The original article has been updated.
