# Supplementary figures and images for: Myelination Increases the Spatial Extent of Analog-Digital Modulation of Synaptic Transmission: A Modeling Study
Source: Front Cell Neurosci. 2020 Mar 3;14:40. doi: 10.3389/fncel.2020.00040 (PMC7063086; doi:10.3389/fncel.2020.00040)

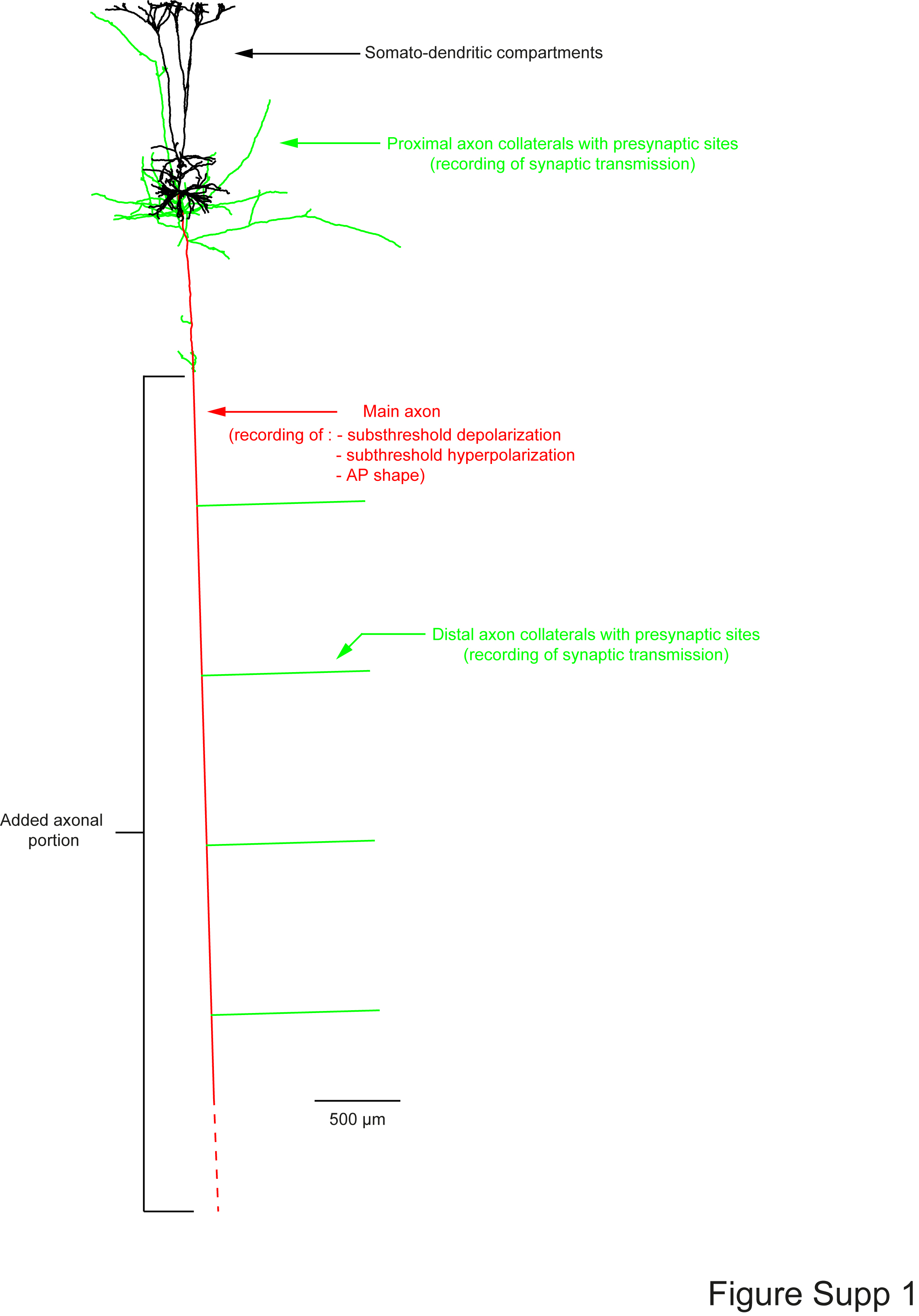

Supplement: FIGURE S1 — Model morphology. Representation of the model morphology based on the reconstructed neuron NMO_07763. Note the axonal portion that has been added to the reconstructed neuron to extend the axonal tree. [file Image_1.JPEG]

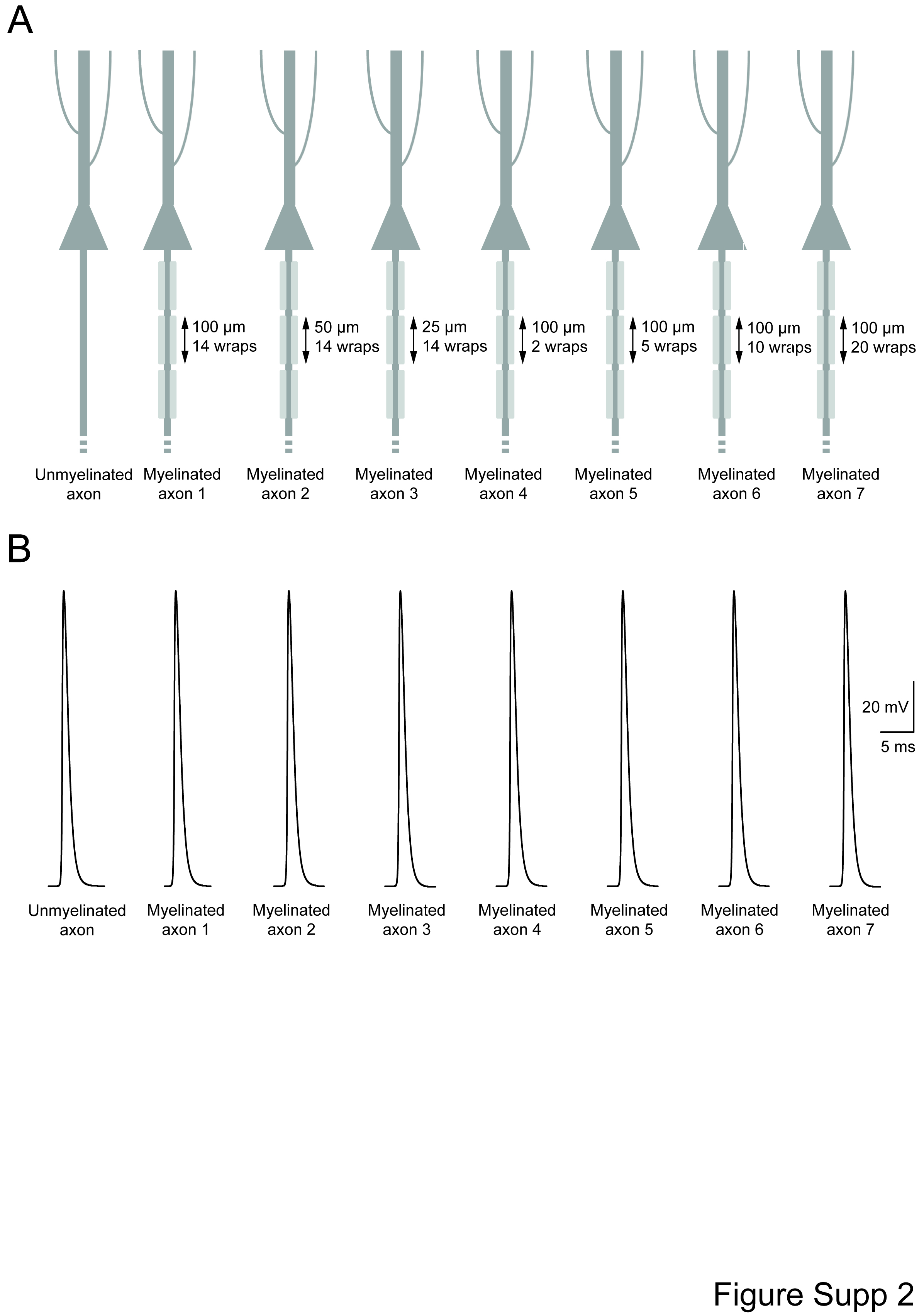

Supplement: FIGURE S2 — Axonal spike waveform at the resting membrane potential is similar in the different simulations. (A) Schematic representation of the different models of the study. (B) Spike waveform at the resting membrane potential (−70 mV) recorded in the middle of the axon (10 mm from the soma) in the different models. Note the similarity of basal axonal spike waveform in the different models. [file Image_2.JPEG]

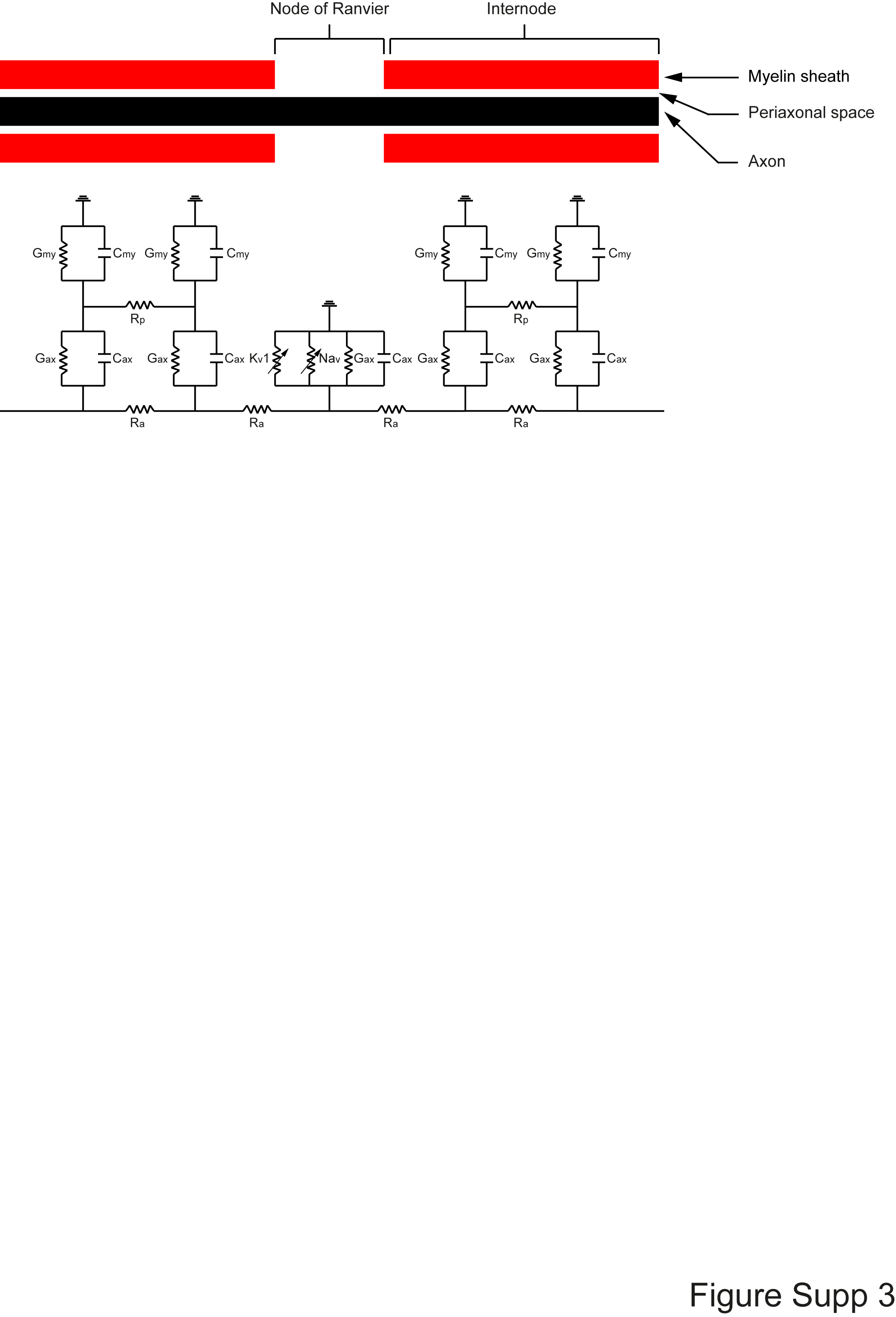

Supplement: FIGURE S3 — Myelin sheath modeling. Up, schematic representation of two internodes and one node of Ranvier. Down, equivalent electric circuit used in the model. Gmy, myelin sheath conductance; Cmy, myelin sheath capacitance; Rp, periaxonal space axial resistance; Gax, axonal passive conductance; Cax, axonal capacitance; Ra, intra-axonal axial resistance; Kv1, Kv1 channels conductance; Nav, axonal Nav channels conductance (Nav1.6 channels conductance). [file Image_3.JPEG]

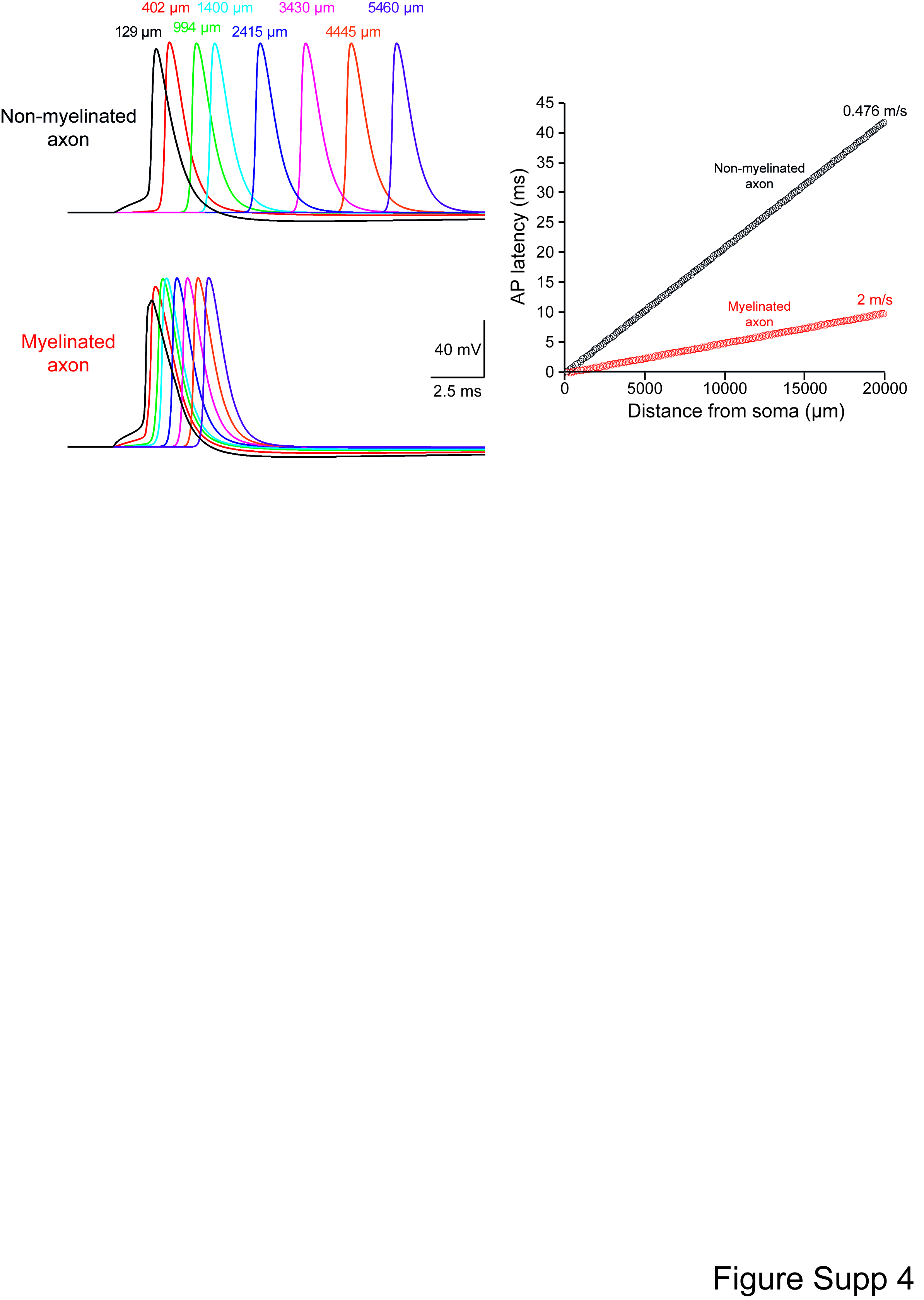

Supplement: FIGURE S4 — Myelination increase velocity of spike propagation into the main axon. Left, voltage traces showing AP propagation along the main axon in the unmyelinated and the myelinated model (Table 2: Myelinated axon 1). Right, plot of the AP latency in the function of the distance from the soma in the myelinated and the unmyelinated model. [file Image_4.JPEG]
